# Supplementary material for: Introduction and validation of a new semi-automated method to determine sympathetic fiber density in target tissues
Source: PLoS One. 2019 May 29;14(5):e0217475. doi: 10.1371/journal.pone.0217475 (PMC6541301; doi:10.1371/journal.pone.0217475)
Supplement: S3 Macro — First macro was used to select peripherin positive areas. (DOCX) [file pone.0217475.s003.docx]

# S3 Macro:

input = getDirectory

output = getDirectory

suffix = ".tif";

processFolder(input);

function processFolder(input) {

list = getFileList(input);

for (i = 0; i < list.length; i++) {

if(File.isDirectory(input + list[i]))

processFolder("" + input + list[i]);

if(endsWith(list[i], suffix))

processFile(input, output, list[i]);

}

}

function processFile(input, output, file) {

print("Processing: " + input + file);

open(input + file);

run("8-bit");

setAutoThreshold("Yen dark");

run("Convert to Mask");

run("Create Selection");

run("ROI Manager...");

roiManager("Add");

run("Color Threshold...");

print("Saved to: " + output);

}
