# Supplementary material for: Introduction and validation of a new semi-automated method to determine sympathetic fiber density in target tissues
Source: PLoS One. 2019 May 29;14(5):e0217475. doi: 10.1371/journal.pone.0217475 (PMC6541301; doi:10.1371/journal.pone.0217475)
Supplement: S4 Macro — A second macro was used to place the ROIs created from the first algorithm over the TH staining images and clearing these selected areas. All TH positive areas that were left were single positive and therefore considered TH positive cells as opposed to fibers. (DOCX) [file pone.0217475.s004.docx]

# S4 Macro:

input = getDirectory

output = getDirectory

suffix = ".tif";

processFolder(input);

function processFolder(input) {

list = getFileList(input);

for (i = 0; i < list.length; i++) {

if(File.isDirectory(input + list[i]))

processFolder("" + input + list[i]);

if(endsWith(list[i], suffix))

processFile(input, output, list[i]);

}

}

function processFile(input, output, file) {

print("Processing: " + input + file);

open(input + file);

roiManager("Select", i);

setBackgroundColor(0, 0, 0);

run("Clear", "slice");

run("Select All");

run("8-bit");

setAutoThreshold("Triangle dark");

//run("Threshold...");

run("Analyze Particles...", "size=72-Infinity circularity=0.20-1.00 display summarize");

print("Saved to: " + output);

}
