# Supplementary figures and images for: Introduction and validation of a new semi-automated method to determine sympathetic fiber density in target tissues
Source: PLoS One. 2019 May 29;14(5):e0217475. doi: 10.1371/journal.pone.0217475 (PMC6541301; doi:10.1371/journal.pone.0217475)

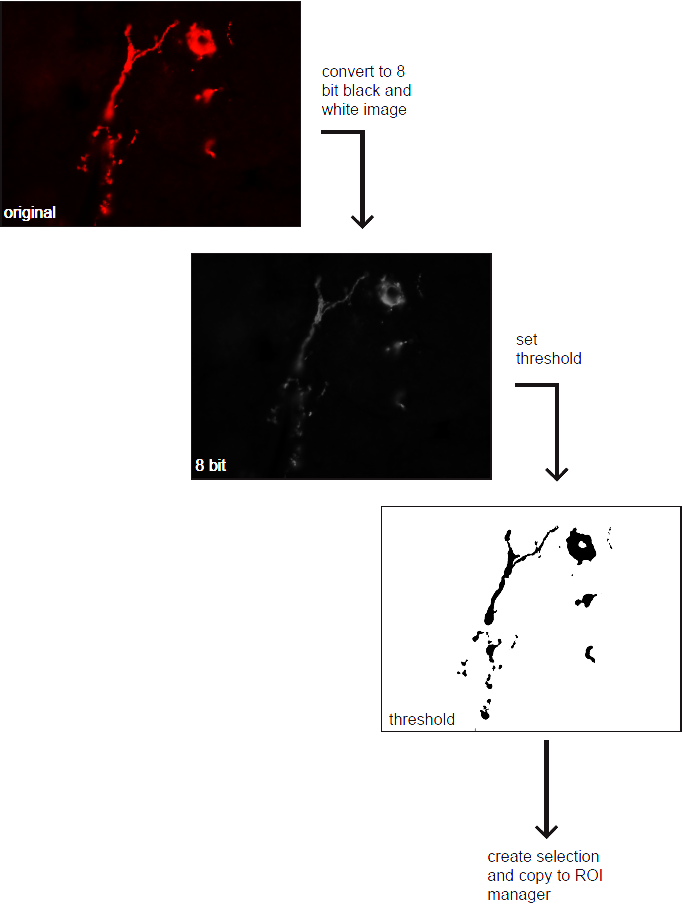

Supplement: S1 Fig — First Images are converted to 8 bit black and white images then a threshold is set for brightness using the Otsu auto threshold tool of ImageJ. Finally a selection was created of the area that was above the threshold and added to the ROI manager tool of ImageJ. (Spleen tissue is shown in this example) (PNG) [file pone.0217475.s005.png]

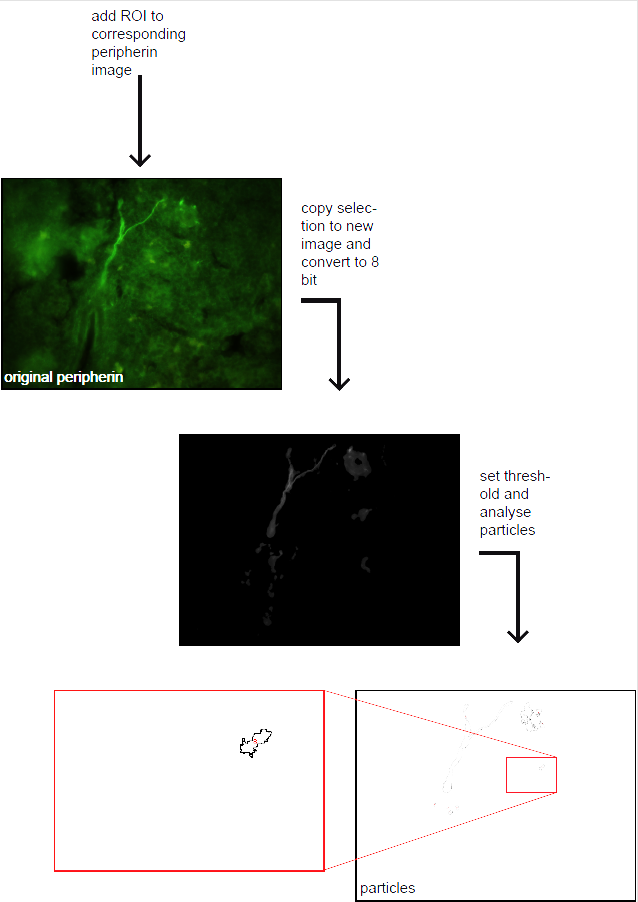

Supplement: S2 Fig — First the ROIs are placed on the peripherin image then a selection is created and copied to a new image. This image is converted to a bit and a threshold is set for brightness using the default auto threshold tool of ImageJ. The areas above the threshold are counted using the analyze particles tool of Image J. (Spleen tissue is shown in this example) (PNG) [file pone.0217475.s006.png]

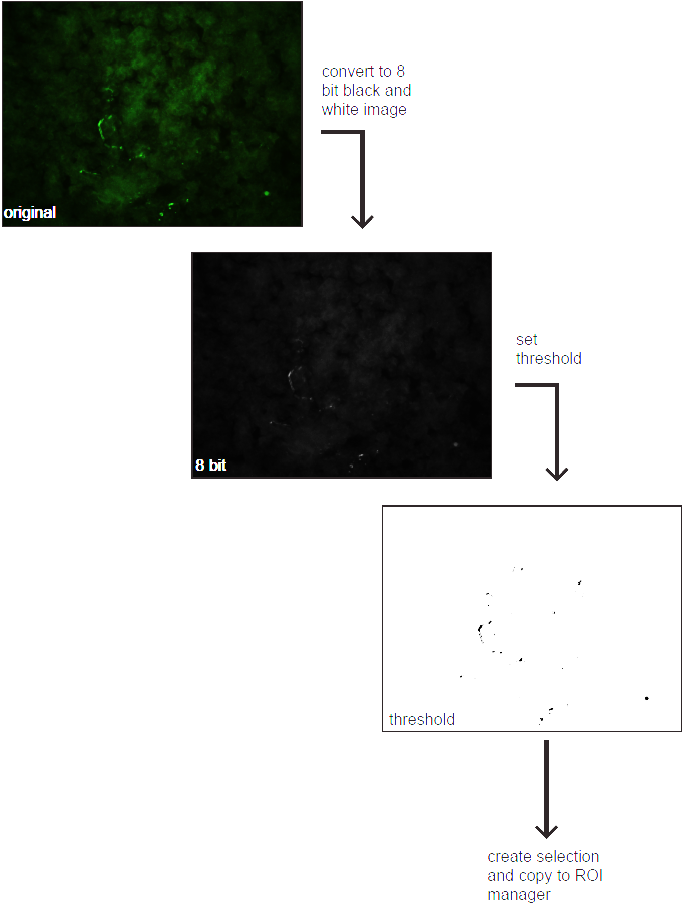

Supplement: S3 Fig — First the images are converted to 8 bit black and white images and a threshold is set for brightness using the triangle auto threshold tool of ImageJ. Then a selection is created of the areas above the threshold and added to the ROI manager tool of ImageJ. (Spleen tissue is shown in this example) (PNG) [file pone.0217475.s007.png]

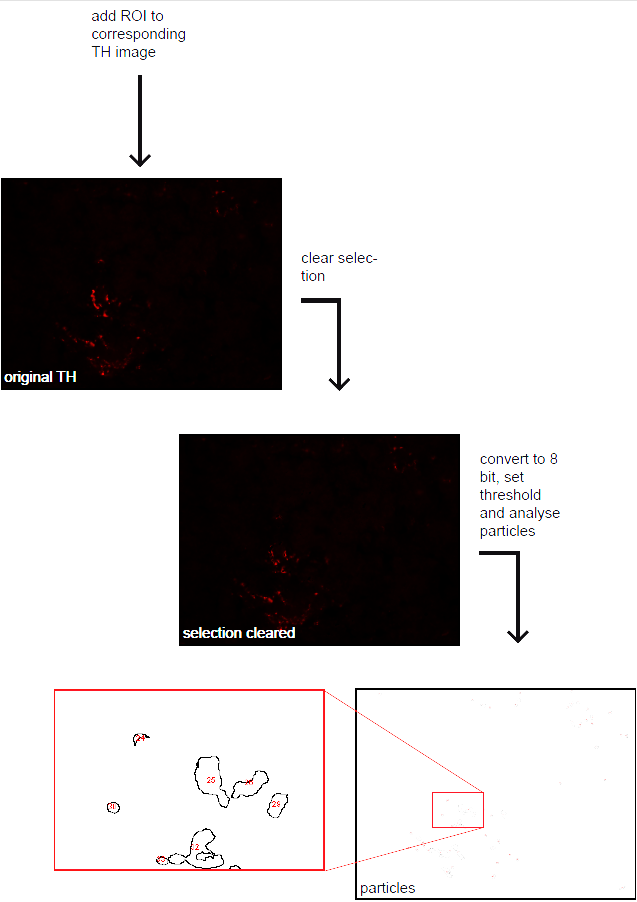

Supplement: S4 Fig — First the ROIs are placed over the TH images then a selection is made of these areas and cleared. The rest of the image is converted to an 8 bit black and white image and a threshold is set for brightness using the triangle auto threshold tool of ImageJ. The “analyze particles” tool is finally used to count particles above the threshold in brightness and above a defined size. (Spleen tissue is shown in this example) (PNG) [file pone.0217475.s008.png]

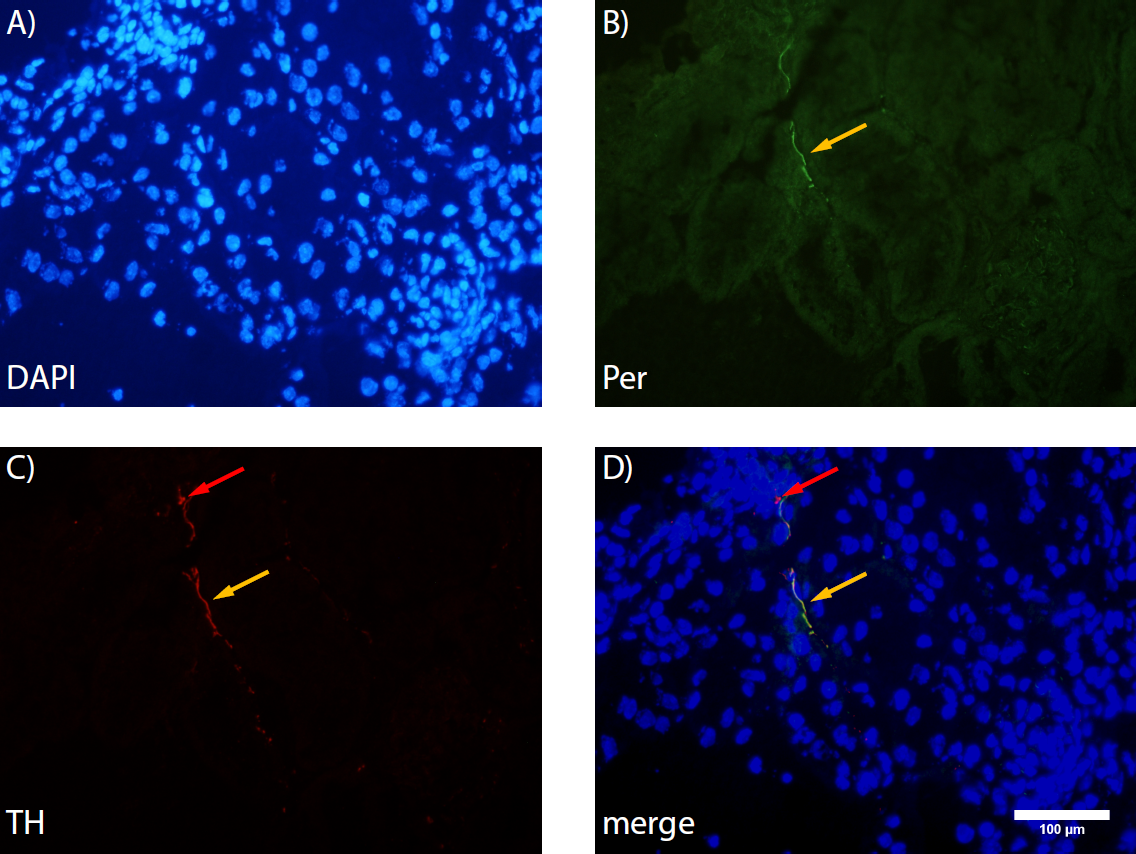

Supplement: S5 Fig — A) Nuclei are labeled with DAPI. B) Peripherin is labeled green (alexa fluor 488). C) TH is labeled red (alexa fuor 594). D) Merged image. Magnification is 400 fold. Red arrows indicate TH positive cells, yellow arrows indicate sympathetic fibers. (PNG) [file pone.0217475.s009.png]

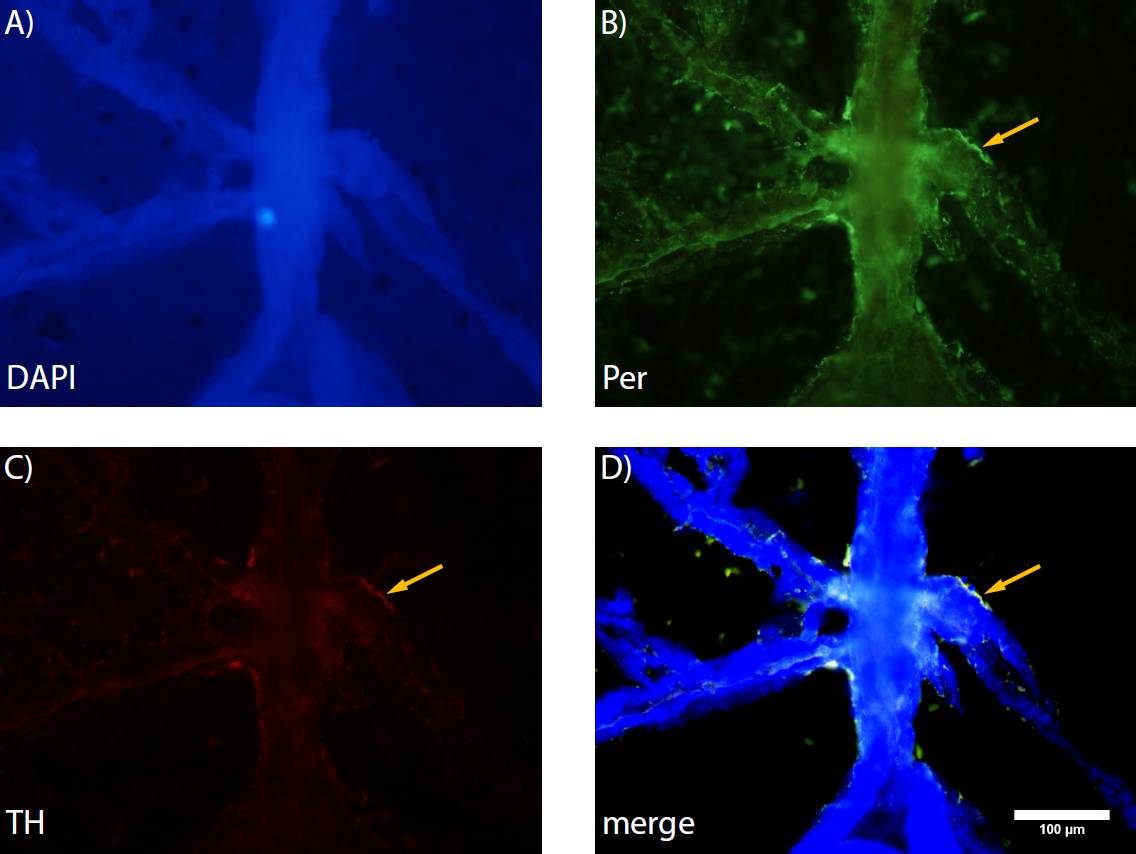

Supplement: S6 Fig — A) Nuclei are labeled with DAPI. B) Peripherin is labeled green (alexa fluor 488). C) TH is labeled red (alexa fuor 594). D) Merged image. Magnification is 400 fold. Yellow arrows indicate sympathetic fibers. (PNG) [file pone.0217475.s010.png]

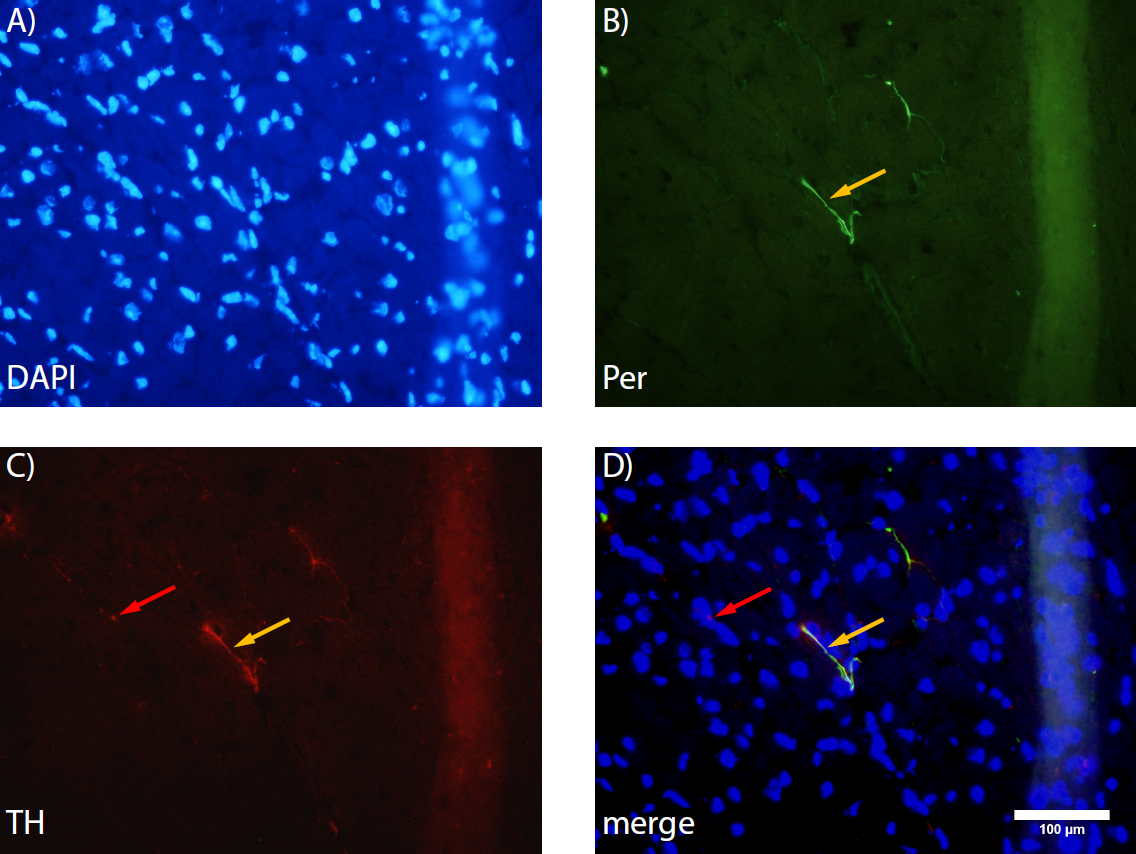

Supplement: S7 Fig — A) Nuclei are labeled with DAPI. B) Peripherin is labeled green (alexa fluor 488). C) TH is labeled red (alexa fuor 594). D) Merged image. Magnification is 400 fold. Red arrows indicate TH positive cells, yellow arrows indicate sympathetic fibers. (PNG) [file pone.0217475.s011.png]

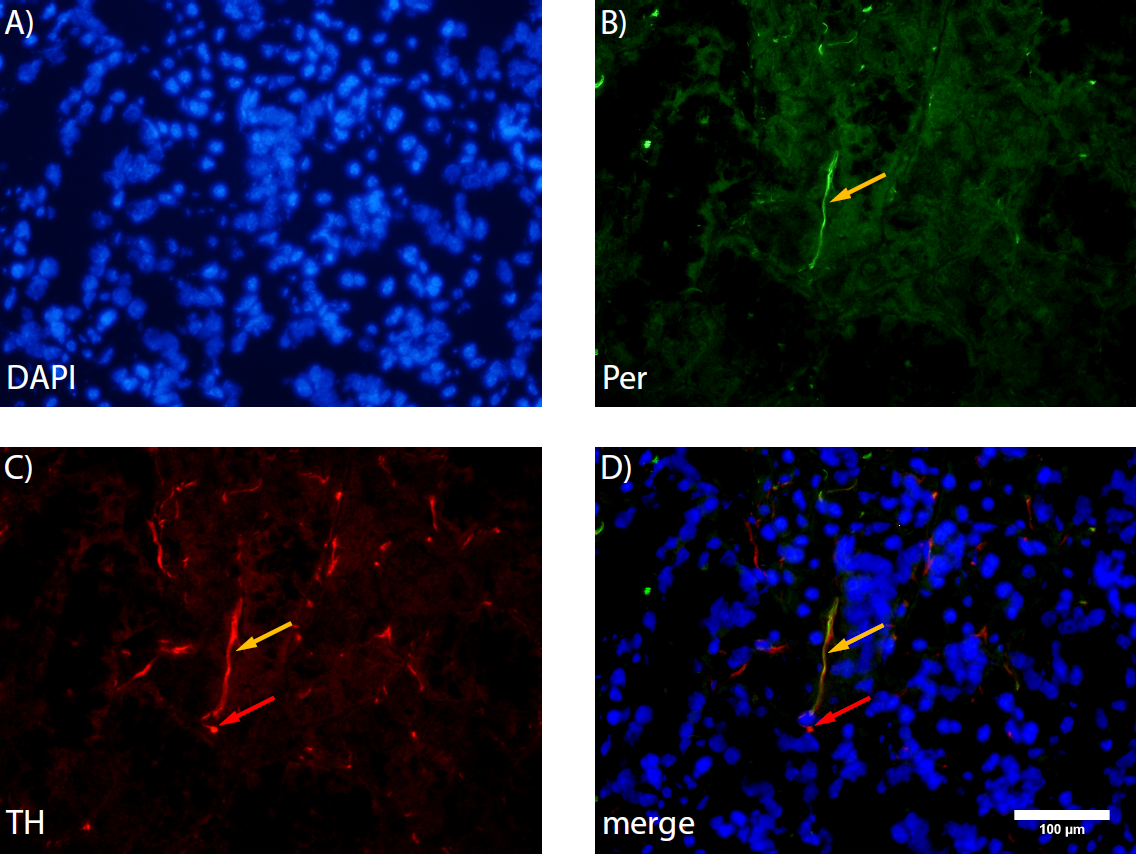

Supplement: S8 Fig — A) Nuclei are labeled with DAPI. B) Peripherin is labeled green (alexa fluor 488). C) TH is labeled red (alexa fuor 594). D) Merged image. Magnification is 400 fold. Red arrows indicate TH positive cells, yellow arrows indicate sympathetic fibers. (PNG) [file pone.0217475.s012.png]

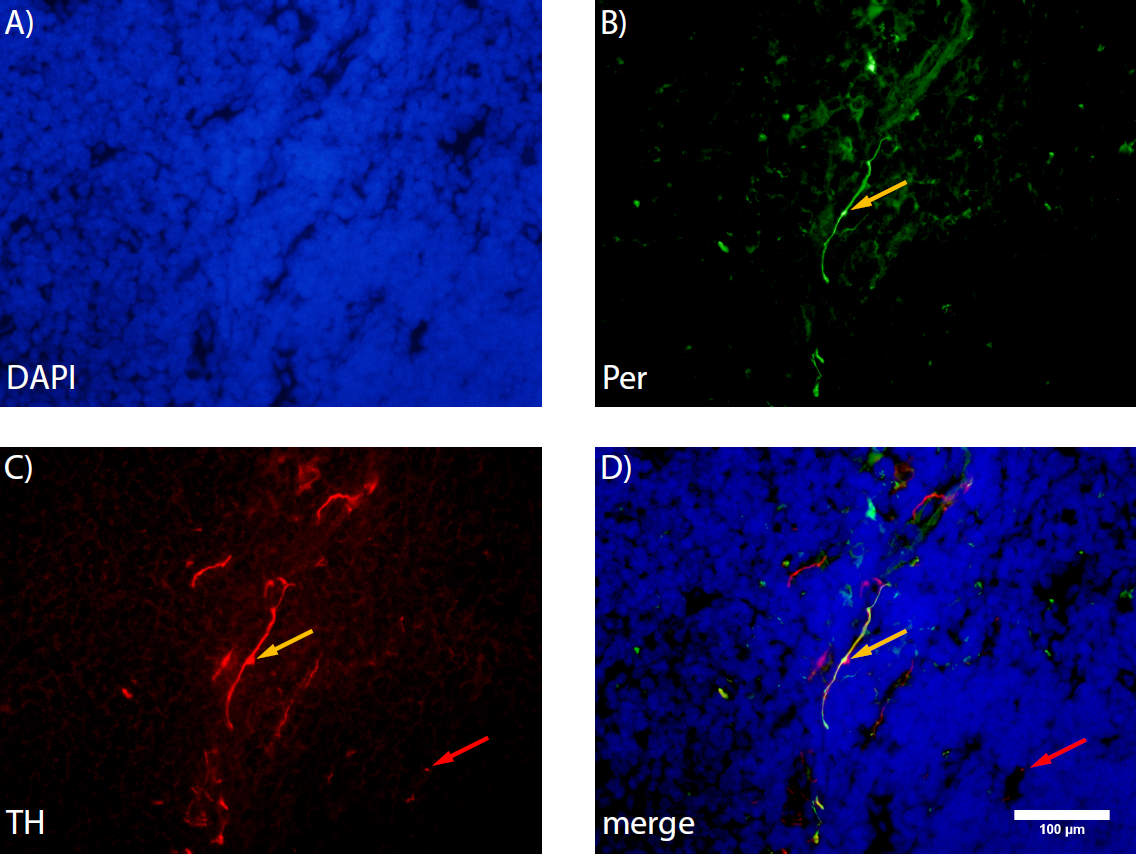

Supplement: S9 Fig — A) Nuclei are labeled with DAPI. B) Peripherin is labeled green (alexa fluor 488). C) TH is labeled red (alexa fuor 594). D) Merged image. Magnification is 400 fold. Red arrows indicate TH positive cells, yellow arrows indicate sympathetic fibers. (PNG) [file pone.0217475.s013.png]
