# Supplementary material for: Introduction and validation of a new semi-automated method to determine sympathetic fiber density in target tissues
Source: PLoS One. 2019 May 29;14(5):e0217475. doi: 10.1371/journal.pone.0217475 (PMC6541301; doi:10.1371/journal.pone.0217475)
Supplement: S1 Table — The CV and SNR values are in the same range for wildtype animals for both methods, but CV and SNR are considerably lower respectively higher with the automated method in SDC3KO animals. (DOCX) [file pone.0217475.s014.docx]

# S1 Table:

| mouse ID | genotype | CV manual | CV automated | SNR manual | SNR automated |
| --- | --- | --- | --- | --- | --- |
| 1 | wildtype | 0.69 | 0.74 | 1.46 | 1.35 |
| 2 | wildtype | 0.52 | 0.45 | 1.92 | 2.21 |
| 3 | wildtype | 0.44 | 0.49 | 2.28 | 2.04 |
| 4 | wildtype | 0.57 | 0.72 | 1.77 | 1.39 |
| 5 | SDC3KO | 0.64 | 0.40 | 1.56 | 2.51 |
| 6 | SDC3KO | 0.57 | 0.22 | 1.74 | 4.55 |
| 7 | SDC3KO | 0.46 | 0.35 | 2.17 | 2.89 |
| 8 | SDC3KO | 0.71 | 0.30 | 1.41 | 3.35 |
